# Recent Advances in Orthodontic Archwires: A Review

**DOI:** 10.7759/cureus.47633

**Published:** 2023-10-25

**Authors:** Priti Chainani, Priyanka Paul, Vinus Shivlani

**Affiliations:** 1 Public Health Dentistry, Sharad Pawar Dental College and Hospital, Datta Meghe Institute of Higher Education and Research (Deemed to be University), Wardha, IND

**Keywords:** orthodontic force, recent advances, bactericide arch wire, robotic wire bending, aesthetic wires

## Abstract

Orthodontic archwires are the primary aid to achieve desirable tooth movement. These wires are also considered to be the backbone of orthodontic treatment. Orthodontic archwires are available in various materials. The journey of advancement of these wires has shown immense growth in aesthetics as well as the mechanical properties of the materials used to ultimately provide patient satisfaction. This review highlights the properties of orthodontic archwires and the disadvantages associated with these wires which limit their use in today's era. The major role of the clinician is to choose the most appropriate alloy as per the needs of the patient. This can be done by accurately analyzing the properties of every material. The introduction of robotic systems in bending archwires and the properties of newer materials like organic polymer wires and bactericide archwires have also been described in this review. Thus, this review article focuses on the recent advances in orthodontic archwires and their properties for selection as per need.

## Introduction and background

The field of orthodontics is evolving every other day. There have been several advances in every aspect including diagnosis, treatment, and retention. Orthodontic archwires have always been an integral part of an orthodontic appliance. During orthodontic treatment, archwires are used as part of fixed appliance to apply force on a tooth. These wires release their energy stored upon placement by producing force with torque over the tooth surface. It has been said that the application of lower forces produces optimum results as compared to heavy forces [1]. Lighter orthodontic forces also lead to the formation of smaller areas of hyalinization. These areas are resorbed easily and within less period of time leading to early tooth movement. The phenomenon behind exerting heavy forces involves increasing the vascular blood pressure and reducing the cellular activity in the periodontium which ultimately slows down the teeth movement for a specific period of time. An advanced quality of wire material which is better for tooth and other oral tissues is needed for an effective orthodontic treatment. Earlier, gold was the first material that was used in making orthodontic archwires. The only drawback with this material was that it was very expensive; hence, not all patients can afford it. Later on, stainless steel became a popular material because it was economical as well as it has better mechanical properties [2]. Its only shortcoming was that it is not aesthetically pleasing which is a concern to patients willing to undergo orthodontic treatment. As the higher age groups are more concerned about aesthetics due to metal braces, archwires were introduced for better patient satisfaction. This led to the introduction of ceramic brackets and wires which gave aesthetically better appearance [1]. The properties of these when compared to stainless steel remain questionable to many clinicians. This urged the introduction of newer materials which combine the aesthetic as well as mechanical properties to yield patient compliance as well as best treatment results [2]. Since there are several options available on the basis of alloys, the choice of material as per the needs of the patient plays a significant role in the final outcome. Hence, clinicians must have adequate information about the material options available and the recent advancements with regard to these orthodontic wires [3].

## Review

Search methodology

**Figure 1:**
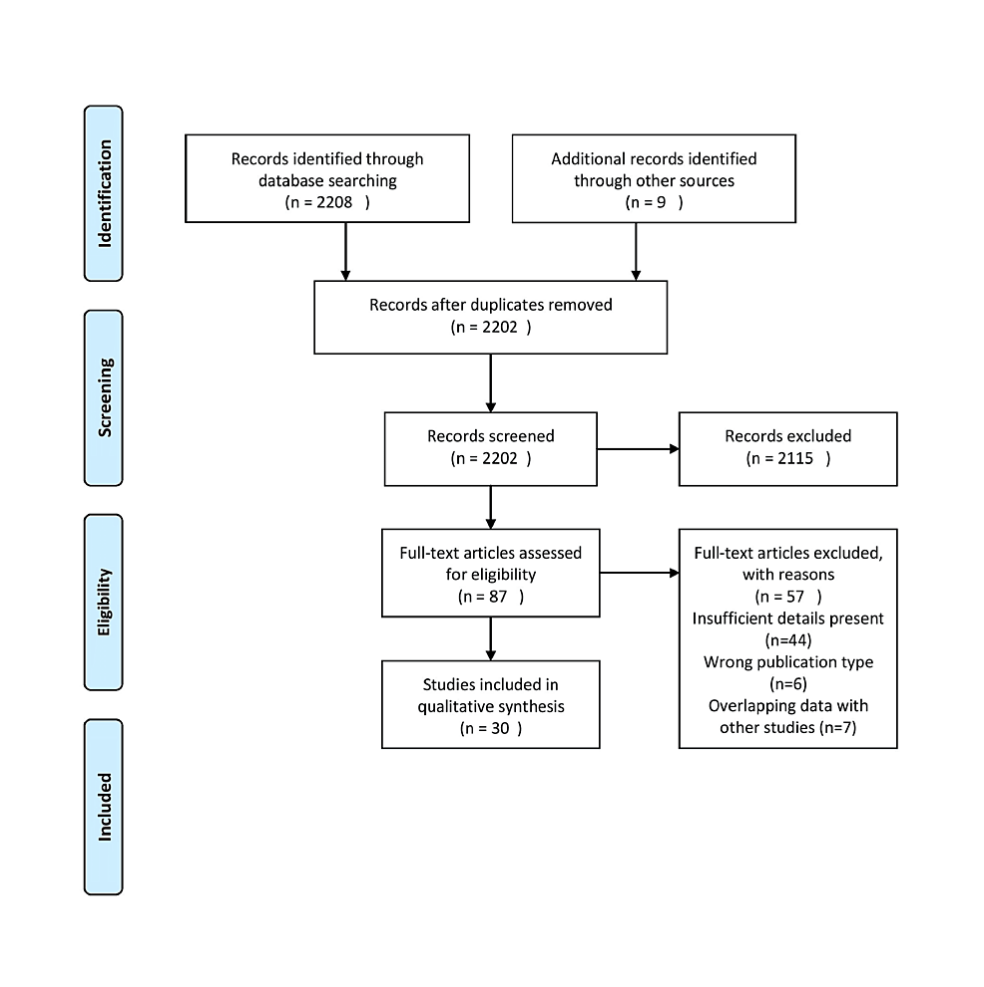
PRISMA flow diagram for search strategy PRISMA: Preferred Reporting Items for Systematic Reviews and Meta-Analysis

We conducted the review through PubMed and Google Scholar in July 2023 using keywords such as "aesthetic wires", "robotic wire bending", "bactericide archwire", "recent advances", and "orthodontic force". In addition, we looked through the bibliographies of pertinent studies for important references. In August 2023, we witnessed an update to the search. The reviewer first checked the titles and abstracts of the retrieved papers against the inclusion and exclusion criteria before checking the complete texts. Both published and unpublished English-language research were taken into consideration for inclusion. Due to resource constraints and the reviewer's inability to access full-text articles, we omitted research and articles that are related to the use of orthodontic archwires and their advances. Figure 1 describes the selection process of articles used in our study.

Properties of archwires

Archwires must have desirable properties that would maximize their use; these include good formability, less stiffness, superior range, good strength, weldability, solderability, and cost-effectiveness [1]. All these ideal requirements cannot be fulfilled by a single archwire material; hence, various materials are available and there are several recent advances, and the specific wire for the desired purpose will produce the best results [2]. Aesthetics has been an integral part of orthodontic treatment. For example, with the introduction of aesthetic brackets, there was a need for aesthetic archwires. With the introduction of new materials, there has been an urge to incorporate improvement in the design, construction methods, and clinical manipulation of the appliances. Hence, the clinician needs to always make a wise decision in the wire selection as well as its clinical manipulation. The newer archwires introduced in orthodontics include aesthetic archwires (composite, Optiflex (Ormco Corporation, Orange, California, United States), and coated), Lee white wires (Lee Pharmaceuticals, Hyderabad/Secunderabad, Telangana, India), Marsenol (Glenroe Technologies, Tallevast, Florida, United States), organic polymer retainer wires, super engineering plastic (SEP) orthodontic wires, nanocoated archwires, orthodontic archwire bending robot system, Motoman UP6 (Yaskawa Motoman, Miamisburg, Ohio, United States), medical-grade titanium wires, SPEED finishing archwires (SPEED System Orthodontics, Cambridge, Ontario, Canada), Nitanium tooth toned archwires (Henry Schein, Melville, New York, United States), Hills Dual-Geometry archwires (Strite Industries, Cambridge, Ontario, Canada), BioForce wires (Orthomax Pty. Ltd, Burwood, Victoria, Australia), new bactericide orthodontic archwires, TiMolium wires (TP Orthodontics, La Porte, Indiana, United States), and Tri-Force wires (Young Specialties, Algonquin, Illinois, United States). The various properties of these advances in archwires are discussed briefly.

Aesthetic archwires

Aesthetics has been an integral part of dentistry these days. Every orthodontic treatment aims to provide the utmost aesthetics to the work. Also, with the evolution of aesthetic brackets and clear aligners, this advancement in archwires has been made. These wires can be of three types: composite, Optiflex, and coated [3].

Composite

The most commonly used is fiber-reinforced composites. These provide several advantages like biocompatibility, less hypersensitivity, high modulus of elasticity, greater yield strength, resilience, and ease of modification [4]. The archwire is available in various diameters and applies light continuous force, and to avoid fractures, sharp bends are avoided [5].

Optiflex

This was introduced by Talass in 1992 [6]. This wire is made of clear optical fiber. It is composed of three layers: silicon dioxide core, silicon resin cladding, and nylon coating. The advantages are as follows: it has a broad range of action and it is capable of applying light forces [6]. Adult patients with aesthetic concerns highly benefit from these wires. Light continuous forces are delivered. Also, flexibility and compatibility with orthodontic brackets are highly efficient with these wires. In contrast, these wires are not economical which limits their use.

Coated

It has been designed to enhance aesthetics and reduce friction. It is done by two conventional air-spray or electrostatic technique. It can be Teflon-coated, epoxy-coated, or Nitanium tooth toned archwires [6]. These wires are affected by masticatory forces and the enzyme activity of the mouth [7].

Lee white wires

These wires were made by Lee Pharmaceuticals. These are resistant stainless steel wires and can be made of nickel titanium (NiTi). They have tooth-colored epoxy coating. This is opaque and serves various other advantages, for example, it does not show discoloration and does not peel off. Moreover, it does not scratch as well [3]. It shows excellent wear resistance, and the color stability of this material is proved to be excellent. This material is very well suitable for ceramic and plastic brackets which ultimately serves superior aesthetics. 

Marsenol

This is designed by Glenroe Technologies. Marsenol has a coating of elastomeric polytetrafluoroethylene emulsion (ETE) wire. These are tooth-colored NiTi wires. The working properties of Marsenol are similar to that of uncoated superelastic NiTi wires [8]. The major advantages of this material are that it is fracture resistant and it remains active for a longer duration of time. Another property of this material is the flexibility of the wire. The material is tooth-colored, hence providing superior aesthetics than conventional orthodontic wires.

Organic polymer retainer wire

This is fabricated from round polyethylene terephthalate and has a diameter of 1.6mm. An orthodontic plier is used to bend an organic polymer retainer wire. The wire has to be heated for few seconds to a temperature of 230 degrees approximately. If this is not done, the wire will return to its original shape [4]. The major purpose of using this wire is to create maxillary retainers. This material is basically for patients who have undergone orthodontic treatment using ceramic and plastic brackets and are extremely conscious regarding aesthetics. 

SEP orthodontic wires

These orthodontic wires provide a better quality of thermal and chemical stability as well as better mechanical strength [9]. In addition, they serve as the best alternative to metallic orthodontic wires due to their excellent metallic properties. These wires are available in various types which are used as per the convenience of the clinician including polyether ether ketone, polyether sulfone, and polyvinylidene difluoride [10].

Nanocoated archwires

Nanoparticles are now the most commonly used element in cases of dry lubricant. These dry lubricants serve the purpose of decreasing the friction between the two contacting surfaces without the use of liquid media. This principle is applied in orthodontics to reduce the friction between the tooth and the wire. These nanoparticles are considered to be dry lubricants which play an important role in reducing the friction between two contacting surfaces [11]. The nanocoated archwires are supplement to any ceramic bracket system which facilitates aesthetics due to its natural tooth color. This also maintains the friction, flexibility, and resiliency of archwires.

Orthodontic archwire bending robot system

Also called the Cartesian-type archwire bending robot system, this system uses SOLIDWORKS software (Dassault Systèmes SOLIDWORKS Corp., Waltham, Massachusetts, United States). The components of this system include the base, bending die, archwire bending system, and turning, feed, and supporting structure of the wire [12]. The robot's end must be flexible in order to do the complex wire bending. Apart from all these advantages, this system has certain shortcomings which limit its use in orthodontics including the accuracy and flexibility of these robots and the constant presence of a human supervision to facilitate accurate work in a complex cavity like the oral cavity [13].

Motoman UP6

It is another type of wire bending robot consisting of an archwire bending actuator and a computer. Advances in this field of robotics are growing everyday and focus much more on human-computer interaction technology. This robot examines several properties like bending properties, kinematics, and archwire spring back [14].

Medical-grade titanium wires

It has been observed that many patients are allergic to nickel, copper, chromium, etc. Hence, to overcome this problem, medical-grade titanium wires are made which are pure titanium alloy and are also considered to be ideal for most sensitive patients. It has two types of alloys, Ti6AL4V and Ti6AL4V extra low interstitial (ELI), which are most commonly used for medical purposes. The wire possesses excellent fracture resistance property which enhances its reliability. Apart from orthodontic purposes, this wire is also used in orthopedic pins and screws, orthopedic cables, ligature clips, surgical staples, joint replacements, and many others [15].

SPEED finishing archwires

It consists of interactions of superelastic spring clip, archwire, and wire slot. The deviation of bracket position relative to archwire results in the deflection of spring clip. This restores energy with recovery. Also, this energy is released gently through precise three-dimensional (3D) tooth positioning [16]. It has a sloping labiogingival shape. This provides ease for the spring clip to interact with the wire slot. The diameters of wires vary depending on the slot size which makes it easy to use and identify. This is because if the clip is misaligned with the wire, the clip bends and stores energy which could be released only with 3D tooth implantation.

Nitanium tooth toned archwires

It is also a type of NiTi archwire. It has superior plastic and friction-decreasing properties. Also, this has tooth-colored coatings which perfectly blend with the natural teeth. It also shows color matching with ceramic, plastic, and composite brackets used for orthodontic treatment. The major disadvantage of this wire is that it is affected by the mastication and the enzyme activity in the oral cavity [17].

Hills Dual-Geometry archwire

This wire is primarily used for posterior segments. It is engineered as an optimal wire for sliding mechanics. An added advantage is that the archwire has extremely superior tensile strength with optimum stiffness. The Hills wire is available in two sizes: 0.018 x 0.01 8 inches with a 0.018-inch round posterior for the 0.018 slot and 0.021 x 0.021 inches with a 0.020-inch round posterior for the 0.022 slot [17,18]. These wires are made from ultra-high-strength stainless steel material, providing excellent stiffness to the wires. 

BioForce wires

This is one of the latest inventions that possess an unusual capacity to alter the transition temperature within the same archwire. The rhodium-plated white appearance provides superior aesthetic properties of this wire. The mechanics behind the function of these archwires are the application of lighter force in the anterior teeth and greater forces on the posterior teeth till the molar plateau is achieved. Hence, the amount of force to be applied can be graded as per the size of teeth. These BioForce wires are considered to be the first wires that are purely organic [19,20].

New bactericide orthodontic archwires

The major problem that is faced by patients seeking orthodontic treatment is the difficulty to maintain oral hygiene. This includes the development of plaque and accumulation of debris around the brackets. This might cause gingivitis which if not treated can further progress to periodontitis. To overcome this, advancement is done in NiTi archwires to make it bactericidal without altering its mechanical properties. This shows the presence of silver nanoparticles within this wire which is proven to reduce the bacterial count by almost 90%. Hence, this can be considered as an excellent alternative to conventional orthodontic archwires for patients who find it difficult to maintain oral hygiene while undergoing orthodontic treatment [21-23].

TiMolium wire

It is made of alpha-beta titanium alloy. This wire combines the properties of stainless steel and titanium wires, that is, the stiffness and ductility of stainless steel and the flexible nature, durability, and long-lasting strength of titanium. It is comprised of an alloy of titanium, aluminum, and vanadium. At varied temperatures, the alpha-beta phases of titanium are stabilized by aluminum and vanadium, respectively. This alloy serves as an excellent combination of properties like strength and surface smoothness. Although stainless steel is most widely used for orthodontic treatment due to its excellent properties, TiMolium can be considered next due to their similar properties and the added advantages of its low modulus and high strength [17,24-26].

Tri-Force wires

**Figure 2:**
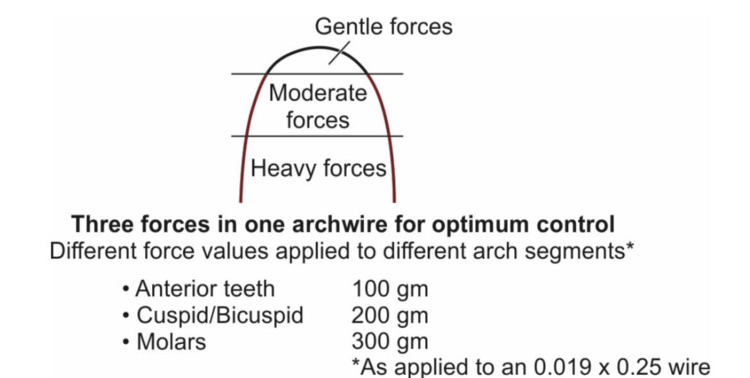
Forces exerted by the Tri-Force wire Reference: [18]

These are special types of wires that are considered to exert varied and desirable amounts of force on a specific tooth region in the oral cavity, that is, firm pressure on the molars, moderate pressure on the premolars, and very gentle pressure on the incisors as shown in Figure 2. Added advantages of this type of archwires are that it prevents the undesirable tilting of the molars and rotation of the premolars and it has a mild impact on the anterior teeth that will not cause pain to patients. Aside from that, the 3D control over these wires is available since the start of treatment [27-30]. 

## Conclusions

Orthodontic archwires have several characteristics such as excellent aesthetic and mechanical properties, durability, and strength which serve as an advantage in their widespread use in orthodontic treatment. The use of these different wires varies depending on the clinician's choice and the needs of the patient. In the 1930s, gold wires were used for orthodontic treatment. Since then, with the advancement in technology, various materials, both biocompatible and with superior qualities, have been introduced which provide ease during the orthodontic treatment. All these advances not only aid in clinician's ease and patient comfort and reduce the chairside time of the patient but also enhance the aesthetic properties, reduce the treatment time, and improve treatment efficiency. As orthodontic wires are a major component of orthodontic treatment, the qualities of these wires play an important role in the treatment result. The orthodontist needs to have a comprehensive understanding of the biomaterials and orthodontic wires to avail the best treatment outcomes in association with patient comfort and satisfaction. The patient must be satisfied with the treatment, and the orthodontist must also make sure that the functionality of the occlusion must be maintained even after the treatment. Occlusal harmony plays an important role in every orthodontic treatment. Hence, these wires must be used judiciously, and the clinician must have adequate knowledge of all the materials and must be updated with their recent advances.
